# Functional Implications of Cathelicidin Antimicrobial Protein in Breast Cancer and Tumor-Associated Macrophage Microenvironment

**DOI:** 10.3390/biom10050688

**Published:** 2020-04-29

**Authors:** Jiawei Chen, Vivian Yvonne Shin, John Chi-Wang Ho, Man-Ting Siu, Isabella Wai-Yin Cheuk, Ava Kwong

**Affiliations:** 1 Department of Surgery, The University of Hong Kong, Pokfulam 999077, Hong Kong; gary0526@hku.hk (J.C.); vyshin@hku.hk (V.Y.S.); cwjohn@connect.hku.hk (J.C.-W.H.); jensiu@hku.hk (M.-T.S.); isacheuk@hku.hk (I.W.-Y.C.); 2 Department of Surgery, The Hong Kong Sanatorium and Hospital, Wan Chai District 999077, Hong Kong; 3 The Hong Kong Hereditary Breast Cancer Family Registry, Shatin 999077, Hong Kong

**Keywords:** breast cancer, cathelicidin antimicrobia, tumor associated macrophage, tumor microenvironment

## Abstract

It is well-established that tumor-associated macrophages (TAMs) play an important role in breast cancer development. Accumulating evidence suggested that human cathelicidin antimicrobial protein (*CAMP*), which is mainly expressed in host defense cells such as macrophages, is crucial not only in combating microorganisms but also promoting tumor growth. Here we report the interaction of *CAMP* with TAMs in breast cancer. *CAMP* expression was upregulated in cancer tissues and in the circulation of breast cancer patients. Surgical removal of tumor decreased CAMP peptide serum level. Knockdown of *CAMP* decreased cell proliferation and migration/invasion ability in breast cancer cells. *CAMP* expression was altered during macrophage M1/M2 polarization and was expressed predominantly in M2 phenotype. In addition, breast cancer cells co-cultured with macrophages upregulated *CAMP* expression and also increased cancer cell viability. Xenograft tumors reduced significantly upon *CAMP* receptor antagonist treatment. Our data implicated that *CAMP* confers an oncogenic role in breast cancer and plays an important role in the tumor microenvironment between TAMs and breast cancer cells, and blocking the interaction between them would provide a novel therapeutic option for this malignant disease.

## 1. Introduction

Breast cancer is the second most common cancer in women, with more than 2 million new breast cancer cases diagnosed in 2018, representing 12% of all new cancer cases worldwide [1]. It was also one of the most leading causes of cancer-related mortalities in women, resulting in more than 600 thousand deaths globally [1]. Accumulating evidence supports that genetic, hormonal, and immunological factors play critical roles in breast cancer development [2,3,4,5].

Human cathelicidin antimicrobial protein (*CAMP*) is the only member of the cathelicidin protein family in humans [6,7]. *CAMP* encodes the human cationic antimicrobial peptide-18 (hCAP-18), which could cleave into different lengths of peptides. LL-37 peptide is the most well-studied peptide [8] that maintained in its pro-peptide form until processed by proteolytic cleavage to bioactive LL-37 [9,10]. *CAMP* is constitutively expressed in different host defense cells, including macrophages, neutrophils, epithelial cells, and endothelial cells, playing important roles not only in combating bacteria, fungi, viruses, and parasites but also regulating various immune functions such as inflammatory reactions, cell proliferation, apoptosis, cell cycle arrest, angiogenesis, and cytokine release [9,10,11,12,13,14,15]. A growing body of evidence illustrated that *CAMP* promoted tumor growth and invasion through angiogenesis initiation and recruitment of immune cells (e.g., monocytes, neutrophils, dendritic cells, mesenchymal stromal cells) [6,16], while promoting wound healing ability and angiogenesis [15]. It has been proposed that LL-37 binds to specific receptors including CXC chemokine receptor type 2 (*CXCR2*), insulin-like growth factor 1 receptor (*IGF-1R*), and purinergic receptor 7 (*P2X7*) in different cell types and tissues, leading to the activation of these receptors [17]. *CAMP* was overexpressed in lung, breast, ovarian, prostate, pancreatic cancer, melanoma, and skin squamous cell carcinoma and facilitated cancer cell growth [6,16,17,18,19,20,21]. These observations suggest that *CAMP* confers a tumorigenic effect in cancers.

Tumor-associated macrophages (TAMs) are the most abundant cells among tumor infiltrated immune cells and have great impact on prognosis [22,23]. Macrophages are regarded as critical effectors during infection, however, accumulating evidence demonstrated a clear role of TAMs in promoting tumor progression [24]. Macrophages are classified into the M1 and M2 phenotypes [25,26]. M1 mainly produce type I pro-inflammatory cytokines, participate in antigen presentation, and are responsible for pro-inflammatory and anti-tumorigenic roles, while M2 produce type II cytokines and have pro-tumorigenic functions [22]. There are markers for each phenotype such as nitric oxide synthase (*iNOS*)for M1, *ARG-1* and *CD163* for M2 [27]. The communication between cancer cells and microenvironment is crucial for disease initiation, development, and progression [28]. In breast cancer stroma, macrophages form the crucial part of tumor microenvironment that occupy more than half of the tumor mass [29,30]. In ovarian cancer, cancer cells induced *CAMP* expression in macrophages to promote tumor progression which indicated an important source of LL-37 [9,11,31,32]. In prostate cancer, overexpression of mouse orthologue cathelicidin-related AMP (*CRAMP*) facilitated early myeloid progenitors into M2 macrophages to promote cancer progression [33]. In contrast, the *CAMP* secreted from M1 macrophages was identified to induce cell death by targeting mitochondria in Burkitt’s lymphoma cells [34]. In view of the intimate relationship between *CAMP* and TAMs, we focused on the characterization of *CAMP* in breast cancer and its interaction with TAMs, which remains largely unknown.

## 2. Materials and Methods

### 2.1. Analysis of TCGA Data

To determine the expression pattern of *CAMP* in breast cancer, the datasets in The Cancer Genome Atlas (TCGA) were used. Briefly, we used Gene Expression Profiling Interactive Analysis, (GEPIA2, http://gepia2.cancer-pku.cn/#index), an interactive web server for analyzing the RNA expression sequencing data (Tumor: *n* = 1085; Normal: *n* = 291) from the GTEx and TCGA projects, based on a standard processing pipeline [35].

### 2.2. Clinical Specimen

Participants were recruited through Queen Mary Hospital, Tung Wah Hospital, and Hong Kong Sanatorium and Hospital through the Hong Kong Hereditary Breast Cancer Family Registry. This study was approved by Institutional Review Board of the University of Hong Kong (UW 15-441). All participants of this study including breast cancer and DCIS patients have agreed and signed the consent form. Patients’ demographic characteristics such as age, histological type, bilateral, staging, metastasis, and histological grade are listed in Table 1.

### 2.3. Cell Culture and Transfection

T47D (ATCC no. HTB-133) were cultured in a 37 °C incubator with RPMI-1640 medium (Invitrogen, NY, USA) supplemented with 10% fetal bovine serum (FBS) and 1% Antibiotic-Antimycotic (Gibco, CA, USA). MCF-7 breast adenocarcinoma cell line (ATCC no. HTB-22) was maintained in DMEM (Gibco) supplemented with 10% FBS and 1% Antibiotic-Antimycotic (Gibco). THP-1 cells (human acute monocytic leukemia cells) were maintained in RPMI-1640 medium (Invitrogen) supplemented with 10% FBS, 1% Antibiotic-Antimycotic (Gibco). Cells were transfected with Allstar Negative Control siRNA and *CAMP* siRNA (Qiagen, CA, USA). Cells were collected after 72 h for further studies.

### 2.4. THP-1 Cell Differentiation into Macrophages

THP-1 cells (2 × 10^5^/mL) were stimulated into undifferentiated macrophages (M0) by incubation with phorbol 12-myristate 13-acetate (PMA, 20 ng/mL) (Sigma-Aldrich) for 3 days, followed by maintaining in complete RPMI-1640 medium for another 3 days. THP-1 cells were treated with A438079 (specific *CAMP* antagonist) at 10 nM dose before adding PMA to stimulate *CAMP*-deficient undifferentiated macrophages. Conditioned medium (CM) collected from MCF-7 was used to stimulate M0 into tumor-associated macrophages (TAMs). CM was centrifuged at 4000 rpm and the supernatant was filtered with Acrodisc^®^ Syringe Filters with Supor^®^ Membrane (Pall Life Sciences, NY, USA). CM was supplemented to 10%FBS before use.

### 2.5. Cell Proliferation Assay

Cells were seeded in a 96-well microtiter plate with triplicates. For gene knockdown experiment, cells were transfected with Allstar Negative Control siRNA or *CAMP* siRNA (Qiagen) using Lipofectamine 3000 (Thermo Fisher Scientific, MA, USA). A438079 was used to treat cells for 5 days aiming to investigate the effect of *CAMP* specific blockade. Cell viability was measured by MTT assay, intracellular purple formazan was solubilized in 100 µL of DMSO followed by the colorimetric product quantified at absorbance 570 nm using a microplate photometer (Thermo Fisher Scientific).

### 2.6. RNA Labelling and Microarray Analysis

Total RNA was extracted from frozen tissues by RNeasy Mini Kit (Qiagen) according to the manufacturer’s instructions. Total RNA was quantified by the NanoDrop ND-1000 and RNA integrity was assessed by standard denaturing agarose gel electrophoresis. Arraystar Human Microarray V3.0 is designed for the global profiling of human protein-coding transcripts, which is updated from the previous Microarray V2.0. About 26,109 coding transcripts can be detected. Sample labelling and array hybridization were performed according to the Agilent One-Color Microarray-Based Gene Expression Analysis protocol (Agilent Technology) with minor modifications. Briefly, mRNA was purified from total RNA after removal of rRNA (mRNA-ONLY™ Eukaryotic mRNA Isolation Kit, Epicentre). Then, each sample was amplified and transcribed into fluorescent cRNA along the entire length of the transcripts without 3’ bias utilizing a random priming method (Arraystar Flash RNA Labelling Kit, Arraystar Inc., Rockville, MD, USA). The labelled cRNAs were purified by RNeasy Mini Kit (Qiagen). The concentration and specific activity of the labelled cRNAs (pmol Cy3/µg cRNA) were measured by NanoDrop ND-1000. Then, 1 µg of each labelled cRNA was fragmented by adding 5 µL of 10 × Blocking Agent and 1 µL of 25 × Fragmentation Buffer, then heated the mixture at 60 °C for 30 min, finally 25 µL of 2 × GE Hybridization buffer was added to dilute the labelled cRNA. A total of 50 µL of hybridization solution was dispensed into the gasket slide and assembled to the expression microarray slide. The slides were incubated for 17 h at 65°C in an Agilent Hybridization Oven. The hybridized arrays were washed, fixed, and scanned using the Agilent DNA Microarray Scanner (part number G2505C).

### 2.7. qRT-PCR

Total RNA was reverse transcribed into cDNA with High Capacity cDNA Reverse Transcription kit (Applied Biosystems). Real-time qPCR was performed using QuantiTect SYBR Green PCR Kit (Qiagen) in LightCycler480 II system (Roche, Rotkreuz, Switzerland). The expression level of housekeeping gene was used for normalization. The reaction for each sample was performed in triplicate. 

### 2.8. Aldehyde Dehydrogenase (ALDH) Activity

Aldehyde dehydrogenase (ALDH) activity was measured by using the ALDEFLUOR™ Assay System (StemCell Technologies, WA, USA) according to the manufacturer’s recommendations. Single cells were resuspended in ALDEFLUOR^TM^ Assay Buffer and incubated with the activated ALDEFLUOR^TM^ Reagent, biodipy-aminoacetaldehyde (BAAA). For negative control, an equal number of cells was also incubated with the activated ALDEFLUOR^TM^ reagent and the specific ALDH inhibitor, diethylaminobenzaldehyde (DEAB). After 40 min of incubation, cells were washed with PBS and resuspended in ALDEFLUOR^TM^ Assay Buffer in 4 °C. Cells were analyzed by a dual laser BD FACS Calibur (BD Biosciences, MA, USA) using CellQuest software. 

### 2.9. Cell Cycle Analysis 

Cells were fixed in 3 mL of ice-cold 70% ethanol at −20 °C overnight. Fixed cells were centrifuged at 1000 g for 5 min. Then the cells were washed by PBS and stained with 1mL of staining solution containing 20 μg/mL of propidium iodide and 0.2 mg/mL of RNase A for 30 min and subjected to flow cytometric analysis. Flow cytometric study was performed by BD FACSCalibur (BD Biosciences) using CellQuest software. The average value of G0/G1, S, and G2/M phase were averaged from at least three independent experiments.

### 2.10. Apoptosis Assay

Cellular apoptosis was detected by FITC Annexin V Apoptosis Detection Kit (BD Pharmingen^TM^, San Diego, CA, USA) according to manufacturer’s instructions. Briefly, cells were suspended in 1× binding buffer at a concentration of 1 × 10^6^ cells/mL. Then, 5 μL of FITC Annexin V together with 5 μL of PI were added to 100 μL of resuspended cells and gently vortexed for 15 min at room temperature in the dark. After incubation, 400 μL of 1× binding buffer was added then analyzed using BD FACSCalibur (BD Biosciences, Woburn, MA, USA).

### 2.11. Migration Assay

Cells were plated into a 12-well cell culture plate and were allowed to reach 90% confluence. A sterile pipette tip was used to scratch the cell monolayer. The scratched area was monitored under microscopy (Olympus CKX41, Waltham, MA, USA) at 0, 6, 24, and 48 h. Images at each time point were acquired by DP controller 3.31.292 (Olympus, MA, USA).

### 2.12. Invasion Assay

Cell invasion assays were performed using the BD BioCoat™ Matrigel™ Invasion Chamber (BD Biosciences, MA, USA) according to the manufacturer’s instructions. Briefly, 1 × 10^4^ cells were resuspended in serum-free medium in the upper chambers, and medium supplemented with 10% FBS served as chemoattractant and was placed in the lower chamber. After 24 h culture at 37 °C, invaded cells were fixed with absolute methanol and stained with crystal violet. Invasive cells were counted at magnification ×200 from six random fields. All experiments were performed in triplicate.

### 2.13. Immunofluorescence Staining

Cells were seeded on the glass coverslips in 24-well plates. After washing with PBS three times, cells were fixed with 2% paraformaldehyde for 15 min at room temperature followed by permeabilization with 0.1% Triton × −100. Afterwards, 3% bovine serum albumin (BSA) was used to block the unspecific binding sites for 50 min at room temperature. Cells were then incubated overnight at 4 °C with the primary antibody: 1:100 diluted anti-CD68 antibody (Abcam, MA, USA), 1:50 diluted iNOS antibody (Santa Cruz Biotechnology, CA, USA), 1:50 diluted ARG-1 antibody (Santa Cruz Biotechnology), 1:50 diluted CAMP antibody (Abcam). Samples were then incubated with secondary antibody: 1:200 diluted Donkey Anti-Rabbit IgG H&L, Alexa Fluor^®^ 594 (Abcam), 1:200 diluted Goat Anti-Mouse IgG H&L (Alexa Fluor^®^ 594 (Abcam) for 50 min at room temperature. After being rinsed with PBS, 4’, 6-diamidino-2-phenylindole (DAPI) was added to stain the cell nucleus and samples were visualized under immunofluorescence microscopy (Nikon, Eclipse 80i, Tokyo, Japan).

### 2.14. Western Blotting

Cell pellets were collected and washed with ice-cold PBS and subsequently lysed in 100 µL of lysis buffer for 20 min. After centrifuged for 15 min at 14,000 g in 4 °C, the supernatant was collected, and the protein concentration was determined by the Bradford assay. Whole cell extracts were fractionated by SDS-polyacrylamide gel electrophoresis (PAGE), and transferred to a polyvinylidene difluoride (PVDF) membrane. After incubation with 5% non-fat milk in TBST to block the unspecific binding sites, membranes were washed with TBST and probed against primary antibodies at 4 °C overnight. Secondary antibodies were either anti-mouse or anti-rabbit conjugated with horseradish peroxidase. Chemiluminescence was determined using ECL Western blotting substrate (Amersham Biosciences).

### 2.15. Development of Stable CAMP Overexpression Cell Lines

Full-length coding sequence of human *CAMP* gene (GenBank accession no. NM_004345) was PCR amplified with primer sequences: TGCATCGAATTCAGGCTGGGCATAAAGGAG (sense) and GCCTGACTCGAGGTAGGGCACACACTAGGAC (anti-sense) and was cloned into pcDNA3.1(+) expression vector. Correct sequence clones were selected and verified with Sanger sequencing. Cells were transfected with *CAMP*-expression plasmid and were selected with 500 µg/mL of G418 for 4 weeks. Cell clones that survived were selected for *CAMP*-overexpression with quantitative RT-PCR.

### 2.16. LL-37 Quantification by Enzyme-Linked Immunosorbent Assay (ELISA)

96-well ELISA plate (Cusabio, Wuhan, China) was pre-coated with antibody specific for antibacterial peptide LL-37. Serum samples from 26 normal healthy individuals, 58 breast cancer patients, or medium from cultured cells were included in this assay. A standard curve was prepared by making serial dilutions of the known concentration of standard. All steps were carried out according to the manufacturer’s instruction. Briefly, 100 µL of serum or culture medium was added into each well and incubated for 2 h at 37 °C. After removing the serum, 100 μL of biotin-antibody (1×) was added and incubated for 1 h at 37 °C. After incubation, each well was washed with wash buffer three times. Then 100 μL of HRP-avidin (1×) was added followed by the washing process. Afterwards, 90 μL of TMB Substrate was added prior to addition of 50 μL of Stop Solution and incubated for 15–30 min at 37 °C with protection from light. The optical density was determined at 450 nm wavelength within 5 min by microplate photometer (Thermo Fisher Scientific).

### 2.17. Transwell Co-Culture Assay

Macrophages differentiated from THP-1 cells were cultured in the absence/presence of MCF-7/T47D cells by using a 0.4 µm transwell system (Sigma-Aldrich). After co-culture for 5 days, macrophages and breast cancer cells were collected for further study.

### 2.18. Immunohistochemistry (IHC) Staining

Tissues were fixed in 4% paraformaldehyde and embedded in paraffin. The tumor was sectioned at 5 μm and was dewaxed and rehydrated by serial immersion in ethanol. After quenched with hydrogen peroxide and treated with sodium citrate, blocking solution was applied to block non-specific binding sites. The slides were subsequently reacted with primary antibody overnight at 4 °C. The slides were then washed with PBS and were incubated with secondary antibodies for 30 min. Sections were visualized by Nikon Eclipse 80i (Nikon) using iView DAB detection kit (Ventana, AZ, USA). Images were acquired by Spot Advanced software.

### 2.19. Animal Study

Four to six weeks old NOD/SCID mice were used for orthotopic injection of cells into the mammary fat pad. Briefly, 0.72 mg of slow release estradiol pellet (Innovative Research of American, Sarasota, FL, USA) was implanted subcutaneously on the neck between the ear and the shoulder of the mice. After 3 days, 2 × 10^6^ T47D cells with empty vector or *CAMP* overexpressing plasmid were injected into mammary fat pad of the mice and the tumor volume was compared after 7 weeks. To further elucidate the role of *CAMP* in cancer cells-TAMs microenvironment, mice were divided into four groups: (i) cancer cells only; (ii) cancer cells + TAMs (cancer cells:TAMs ratio = 5:1); (iii) cancer cells + *CAMP* antagonist A438079 (35 mg/kg); and (iv) cancer cells + TAMs + *CAMP* antagonist A438079. A438079 was administered intraperitoneally per week in dose of 35 mg/kg. Tumor volume was calculated using the formula (length × width^2^)/2. All experiments were conducted under to the approval of Committee on the Use of Live Animals in Teaching and Research (CULATR) of The University of Hong Kong Li Ka Shing Faculty of Medicine (4341-17).

### 2.20. Statistical Analysis

The differences between groups were estimated by Student’s *t*-test, and non-parametric Mann–Whitney U test as appropriate in calculations. *p* < 0.05 was considered as statistically significant. All experiments were performed in triplicate. The statistical analyses were performed using GraphPad Prism 6.0 (GraphPad Software Inc., CA, USA). 

## 3. Results

### 3.1. CAMP mRNA and Cathelicidin Antimicrobial Peptide Are Upregulated in Breast Cancer

**Figure 1:**
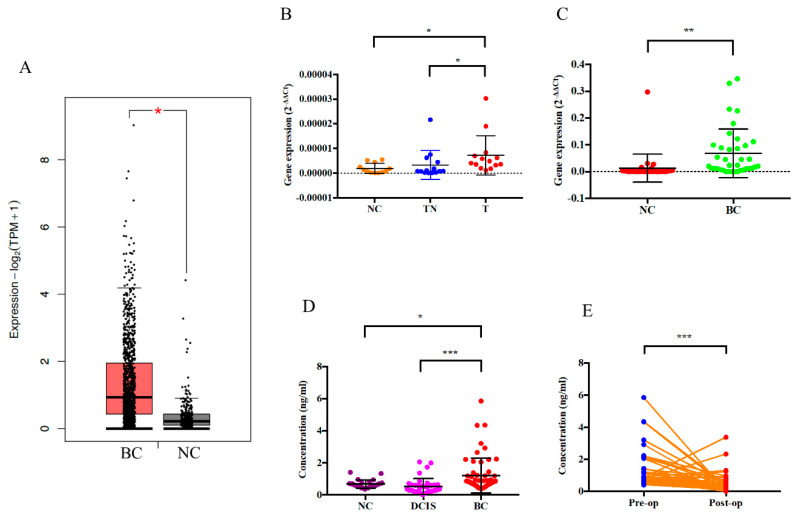
*CAMP* mRNA and cathelicidin antimicrobial peptide is upregulated in breast cancer. (**A**) *CAMP* expression data in normal controls and breast cancer tumor tissues from The Cancer Genome Atlas (TCGA) database; (**B**) *CAMP* mRNA expressions in normal controls, breast cancer patients’ tumor tissues and tumor adjacent normal tissues; (**C**) *CAMP* mRNA expression in normal controls and breast cancer patients’ plasma; (**D**) serum concentration of peptide LL-37 in normal controls, DCIS, and breast cancer patients; (**E**) serum LL-37 levels of pre-/post-operation breast cancer patients. * *p* < 0.05, ** *p* < 0.01, *** *p* < 0.001 indicates statistically different. (NC: normal control; BC: breast cancer; TN: tumor adjacent normal tissue; T: breast cancer tissue).

To identify the dysregulated genes in breast cancer, we selected three pairs of tumor and tumor adjacent normal tissues for microarray analysis. With cut-off of 2-fold, we found that *CAMP* is one of the most upregulated genes in breast cancer which was further confirmed by qRT-PCR. First, we analyzed the *CAMP* mRNA expression between breast cancer (BC) tissues and normal tissues (NC) using the publicly available TCGA database. The result showed that the expression of *CAMP* was upregulated significantly in the cancer tissues compared to the normal tissues (Figure 1A). Then, we further validated in our cohort that the expression level of *CAMP* was higher in breast cancer patients’ tissues (T) when compared to their paired normal tissues (TN) and normal control tissues (NC) (Figure 1B). Additionally, we revealed *CAMP* was upregulated in breast cancer patients’ plasma when compared with normal individuals (Figure 1C). The concentration of human antibacterial peptide LL-37 was significantly higher in breast cancer patients than in normal individuals and ductal carcinoma in situ (DCIS) cases (Figure 1D). Among the breast cancer patients, we observed a decreased LL-37 level in post-operative blood samples (Figure 1E).

### 3.2. CAMPConfers Oncogenic Roles in Breast Cancer

**Figure 2:**
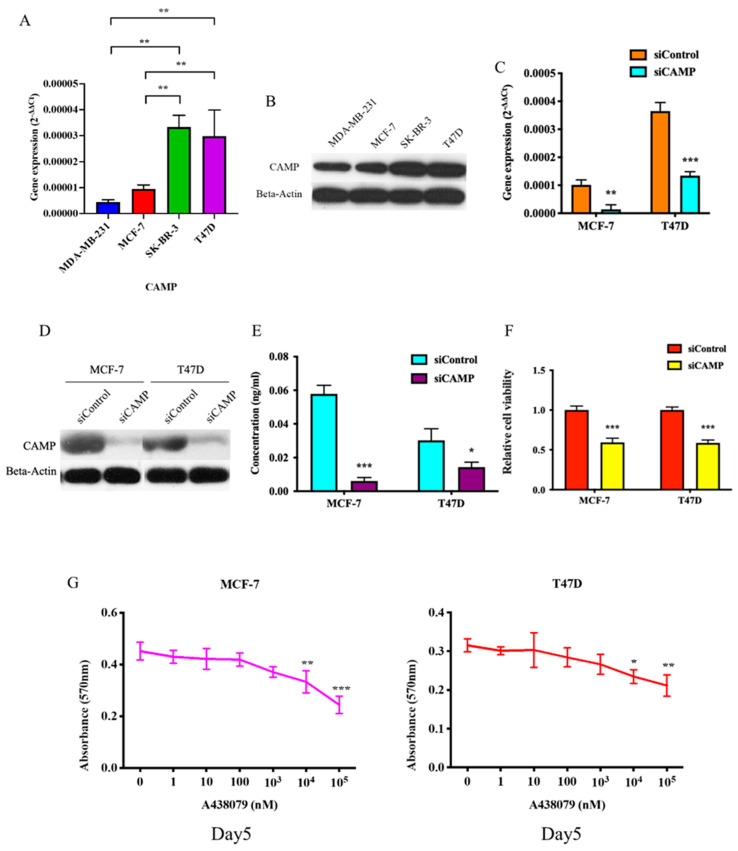
*CAMP* inhibition significantly decreased cell viability in breast cancer cells. (**A**) *CAMP* mRNA expression and protein level (**B**) in breast cancer cell lines; qRT-PCR (**C**) and Western blotting results (**D**) showed that *CAMP* was significantly suppressed by siRNA; (**E**) secreted *CAMP* peptide level in cell culture medium was detected by ELISA in breast cancer cells; (**F**) MTT assay after *CAMP* siRNA in breast cancer cells; (**G**) *CAMP* receptor specific antagonist, A438079inhibited cell proliferation. * *p* < 0.05, ** *p* < 0.01, *** *p* < 0.001 indicates statistically different.

**Figure 3:**
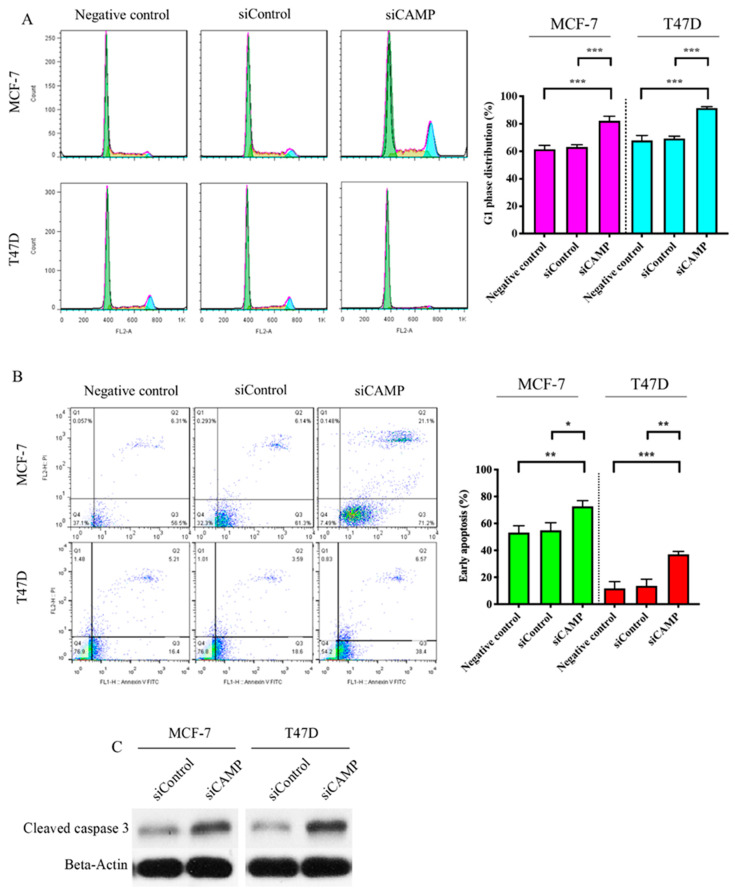
*CAMP* knockdown altered cell cycle progression and apoptosis. (**A**) Significantly increased G1 phase arrest was observed in both cell lines after *CAMP* knockdown (negative control: parental breast cancer cells); (**B**) early apoptotic cells were induced upon *CAMP* siRNA (negative control: parental breast cancer cells); (**C**) Western blot result of cleaved caspase-3 in breast cancer cells after *CAMP* knockdown. * *p* < 0.05, ** *p* < 0.01, *** *p* < 0.001 indicates statistically different.

**Figure 4:**
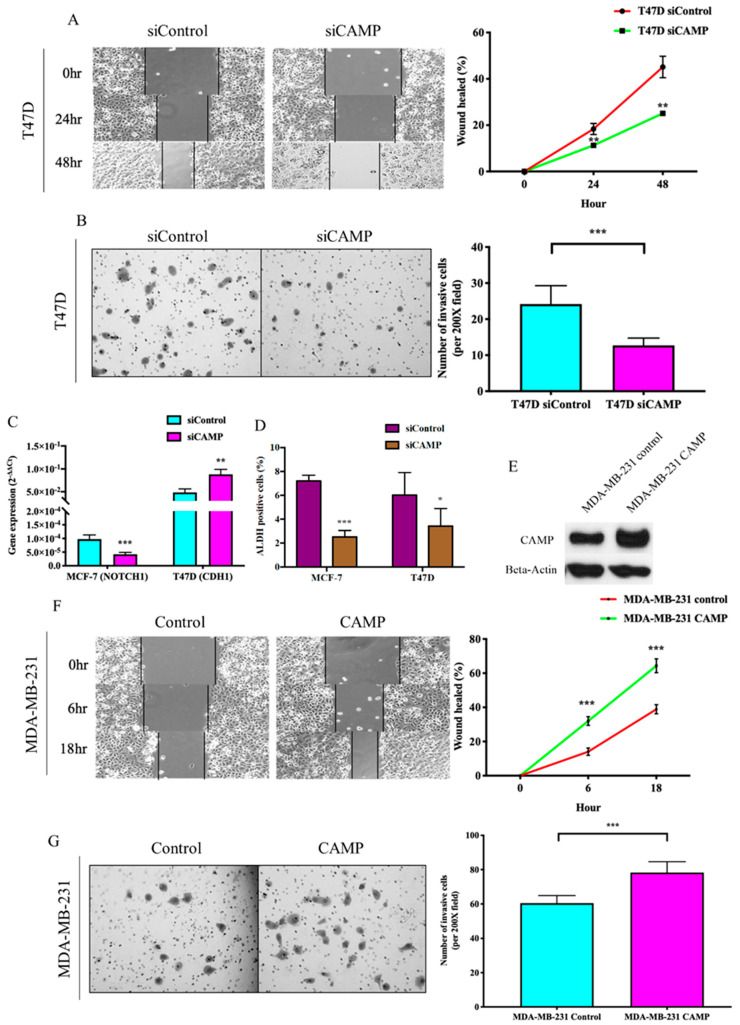
*CAMP* played an essential role in maintaining aggressiveness and stemness in breast cancer cells. (**A**) Wound healing ability was inhibited by *CAMP* siRNA; (**B**) invasive cells were decreased by knocking down of *CAMP*; (**C**) *NOTCH1* expression was altered by *CAMP* knockdown; (**D**) aldehyde dehydrogenase (ALDH)-positive cell population was significantly decreased by *CAMP* siRNA treatment in both breast cancer cell lines; (**E**) Western blot result of *CAMP* protein in stable MDA-MB-231 *CAMP* overexpressing cells; wound healing ability (**F**) and invasive ability (**G**) were significantly enhanced by ectopic *CAMP* expression in MDA-MB-231 cells. * *p* < 0.05, ** *p* < 0.01, *** *p* < 0.001 indicates statistically different.

To further elucidate the functional implication of *CAMP* in breast cancer, we investigated the expression of *CAMP* in different breast cancer cell lines (MDA-MB-231: triple-negative breast cancer; MCF-7: estrogen receptor-positive; SK-BR-3: HER2-positive; T47D: estrogen receptor-positive) by qRT-PCR (Figure 2A) and Western blotting (Figure 2B). Knockdown of *CAMP* decreased mRNA expression, protein level, and secreted *CAMP* protein (Figure 2C–E). MTT assay result confirmed that si*CAMP* led to significant growth inhibition in MCF-7 and T47D cell lines (Figure 2F) but not in SK-BR-3 (data not shown). Treatment with *CAMP* receptor-specific antagonist, A438079, dose-dependently inhibited breast cancer cell growth (Figure 2G). Cell cycle analysis suggested a significant G1 phase arrest in *CAMP*-deficient cells (Figure 3A). *CAMP* knockdown also led to robust increase in early apoptotic cells (Figure 3B) along with increased cleaved Caspase 3 expression (Figure 3C). Both cell migration ability (Figure 4A) and invasiveness (Figure 4B) were suppressed upon *CAMP* knockdown in T47D cells, which may be due to increased *CDH1* and decreased ALDH activity (Figure 4C,D). To consolidate this result, we developed stable *CAMP* overexpressing MDA-MB-231 cells (Figure 4E) and found that ectopic *CAMP* expression enhanced migration and invasion ability (Figure 4F,G).

### 3.3. CAMP is Essential in Tumor Cells-M2 Macrophage Microenvironment

**Figure 5:**
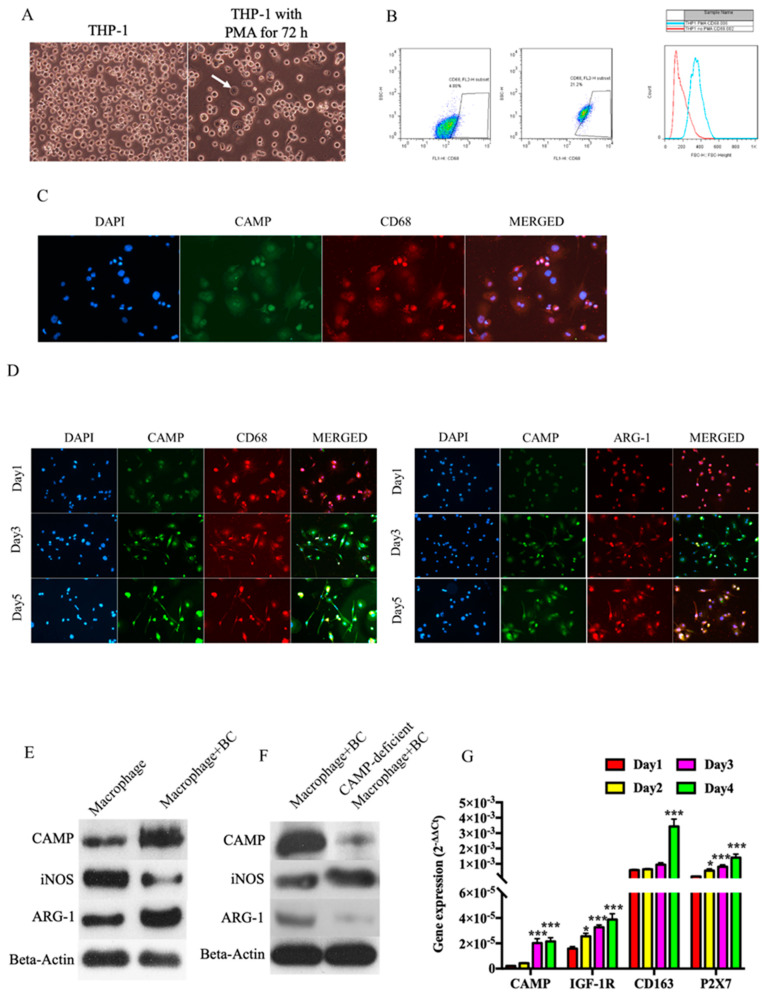
*CAMP* played an important role in the transition from M0 to M2 macrophages. (**A**) Non-specific macrophages were differentiated from THP-1 cells by phorbol 12-myristate 13-acetate (PMA) treatment; (**B**) flow cytometry result showed that CD68+ve cells population increased upon PMA treatment; (**C**) immunofluorescence (IF) staining of CD68 and CAMP in differentiated macrophages from THP-1 cells; (**D**) IF staining of CAMP, CD68, and ARG-1 in macrophage co-cultured with breast cancer cells; (**E**) Western blotting showed that iNOS decreased while *ARG-1* and CAMP increased in macrophage during co-culture with breast cancer cells; (**F**) ARG-1 protein expression decreased and iNOS decreased in CAMP-deficient macrophages co-cultured with breast cancer cells compared to control group; (**G**) mRNA expression levels of *CAMP*, M2 marker *CD163*, *CAMP*-specific receptor *P2X7*, and *IGF-1R* were increased in macrophages cultured in conditioned medium. * *p* < 0.05, *** *p* < 0.001 indicates statistically different.

THP-1 cells were differentiated into macrophage (M0) by incubation with PMA for 72 h (Figure 5A). *CD68*, which is a marker for macrophages, were increased upon PMA treatment by flow cytometry analysis (Figure 5B). Immunofluorescence identified *CD68* expression was co-expressed with *CAMP* in macrophages (Figure 5C). After macrophages were co-cultured with T47D cells in the transwell system, immunofluorescence staining showed increased M2 marker (*ARG-1)* and *CAMP* immunofluorescence intensity (Figure 5D), while expression of M1 marker (*iNOS)* was decreased (Figure 5E). To further delineate the role of *CAMP* in the transition to M2 macrophages when co-cultured with breast cancer cells, we used A438079 pre-treated THP-1 cells for macrophage differentiation, aiming to block the effect of *CAMP* during this process. In the *CAMP*-deficient macrophage group (A438079 pre-treated group), M2 marker *ARG-1* expression was decreased when compared to the control group co-culture with breast cancer (BC) cells (Figure 5F). In addition, expressions of *CAMP* M2 marker (*CD163), P2X7* (*CAMP*-specific receptor), and *IGF-1R* increased in a time-dependent manner (Figure 5G). 

**Figure 6:**
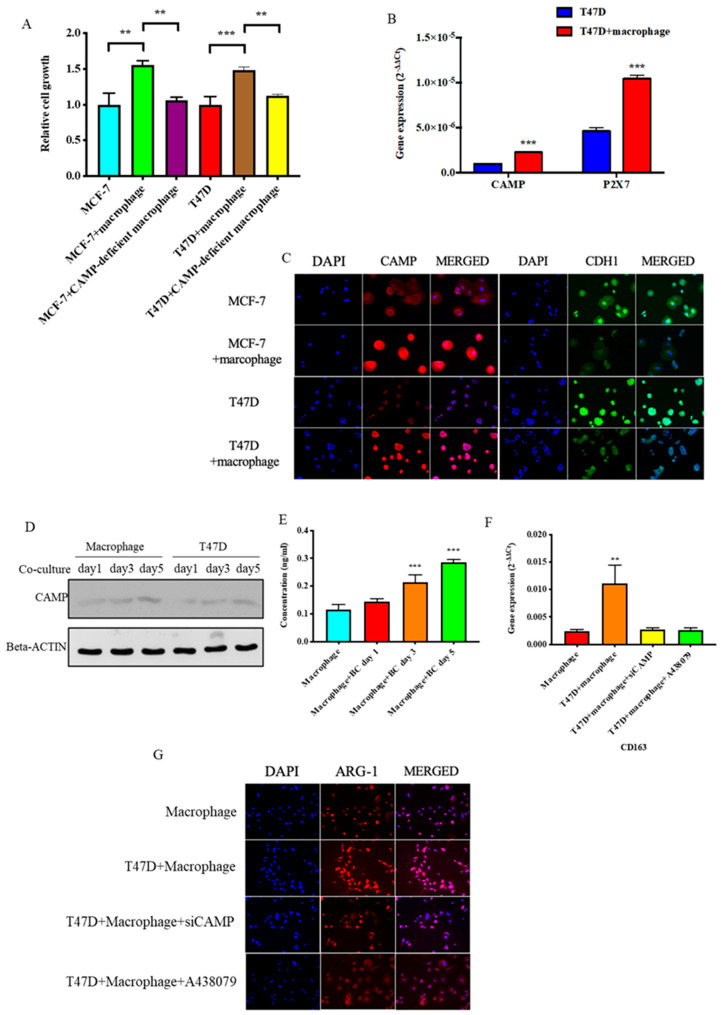
*CAMP* contributed in the interaction between tumor-associated macrophages (TAMs) and cancer cells. (**A**) Cell proliferation ability was significantly enhanced in both breast cancer cells after co-culture with TAMs but not with *CAMP*-deficient macrophages; (**B**) both *CAMP* and its receptor *P2X7* expressions were upregulated in breast cancer cells after co-cultured with TAMs; (**C**) IF staining showed increased immunofluorescence intensity of CAMP and decreased intensity of CDH1 in breast cancer cells co-cultured with TAMs; (**D**) Western blot analysis showed CAMP protein level increased with time in breast cancer cells co-cultured with macrophages; (**E**) secreted CAMP protein level was detected by ELISA in co-culture medium of macrophages and breast cancer cells; (**F**) qRT-PCR and (**G**) IF staining showed that upregulation of ARG-1/CD163 induced in macrophages after co-culture with cancer cells was abrogated by *CAMP* knockdown. ** *p* < 0.01, *** *p* < 0.001 indicates statistically different.

To further mimic the microenvironment between breast cancer cells and TAMs, we indirectly co-cultured breast cancer cells with macrophages in a 0.4 μM transwell system. Co-culture of breast cancer cells with M2 macrophages promoted cancer cell proliferation and was abated in the cancer cells co-cultured with *CAMP*-deficient macrophages (Figure 6A) with increased *CAMP* and *P2X7* expressions (Figure 6B). IF staining revealed *CAMP* signal intensity was upregulated while *CDH1* intensity was decreased in the presence of M2 macrophages (Figure 6C). The Western blotting result showed *CAMP* protein expression increased in both macrophages and breast cancer cells after co-culture for 5 days (Figure 6D). *CAMP* peptide concentration was increased in medium during co-culture of breast cancer cells and macrophages (Figure 6E). The induced M2 marker *(CD163* and *ARG-1*) expressions in macrophages during co-culture with T47D were impeded by *CAMP* knockdown or antagonist treatment, respectively (Figure 6F,G).

### 3.4. CAMPInhibition Reduced Tumor Growth In Vivo

**Figure 7:**
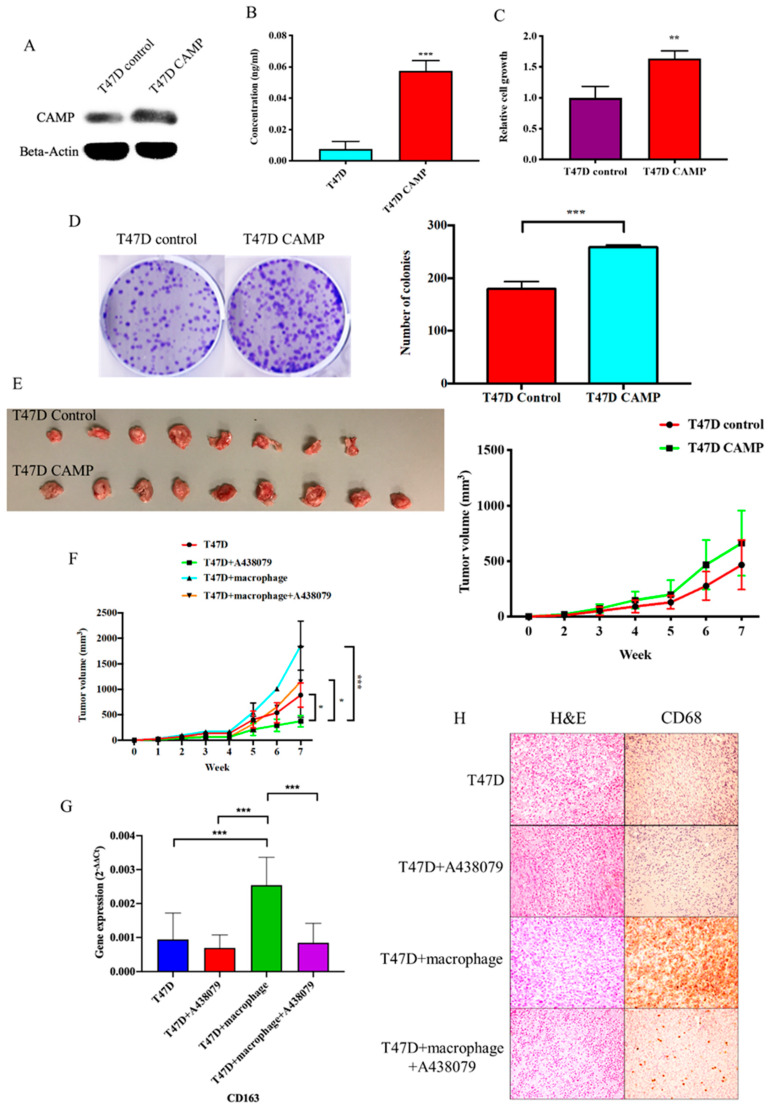
*CAMP* is essential for the tumor promoting effect of TAMs in vivo. (**A**) Western blotting confirmed CAMP is upregulated in stable T47D *CAMP* overexpressing cells; (**B**) secreted CAMP protein level was higher in *CAMP* overexpressing cells’ culture medium than in the control group; (**C**) *CAMP* overexpression increased cell viability in breast cancer cells; (**D**) more colonies were observed in the *CAMP* overexpressing group than the control group; (**E**) *CAMP* overexpression groups exhibited larger tumor volumes than the control group; (**F**) the tumor promoting effect of TAMs was abolished by A438079; (G) *CD163* mRNA expression in mice tissues; (H) IHC staining of human CD68 expression in mice tumors. * *p* < 0.05, ** *p* < 0.01, *** *p* < 0.001 indicates statistically different.

To further investigate the role of *CAMP* on tumor growth, we developed a *CAMP*-stable overexpressing breast cancer cell line (Figure 7A). Secreted *CAMP* protein level was upregulated in *CAMP* overexpressing cells when compared to the control group (Figure 7B). MTT and colony formation assay enhanced cell growth ability in *CAMP* overexpressing cells when compared to vector control (Figure 7C,D). In the animal study, we found that mice with *CAMP* overexpressing cells had larger tumor volume than vector control, suggesting an oncogenic role of *CAMP in vivo* (Figure 7E). Additionally, mice with cancer cells + macrophages exhibited the largest tumor volume while the *CAMP* receptor antagonist inhibited the tumor growth (Figure 7F). The IHC staining result showed high expression of human-specific CD68 in the T47D + macrophages group, which was diminished in the T47D + macrophage + A438079 group. Besides, human-specific CD68 was barely seen in T47D and T47D + A438079 groups (Figure 7G).

## 4. Discussion

To date, around 30 cathelicidin family members have been identified in mammals. However, there is only one cathelicidin *CAMP*, namely hCAP-18, that has been identified in humans [36,37]. LL-37 is a C-terminal peptide that gains its activity after being proteolytically cleaved from the pro-form hCAP-18 [38,39,40]. *CAMP* was first identified as an endogenous antibiotic due to its broad spectrum antimicrobial activity to eliminate Gram-positive and Gram-negative bacteria, viruses, and fungi by disrupting their membranes [41]. *CAMP* is constitutively expressed in neutrophils, natural killer (NK) cells, dendritic cells, monocytes, and especially macrophages [42,43,44,45]. It has been widely accepted that *CAMP* has an important role in inflammatory-related diseases such as chronic inflammatory skin disease [38], Crohn’s disease [46], and autoimmune diseases including psoriatic arthritis [47], systemic lupus erythematosus [48], and atherosclerosis [49]. Apart from its antimicrobial function, *CAMP* has also been reported to regulate a wide range of activities including apoptosis, cell proliferation, angiogenesis, and wound healing [37,50]. For example, *CAMP* was also known to be upregulated in breast cancer tissues compared to normal tissues [51]. In our study, *CAMP* was upregulated in both cancer tissues and circulation. LL-37 peptide level was not significantly different between normal and pre-cancerous DCIS individuals, but obviously higher in breast cancer patients, suggesting its oncogenic role in cancer progression. The decreased serum concentration of LL-37 after surgical removal of tumor indicated its potential as a circulating biomarker for breast cancer. 

*CAMP* was reported to exert its cancer-related roles by regulating cell proliferation, invasion, migration, apoptosis, and cell cycle [52]. There was a study that reported that LL-37 binds to ErbB2 receptor and transient receptor potential cation (*TRPV2*) which led to activation of *MAPK* signaling and the *PI3K/AKT* pathway in breast cancer cells and resulted in enhanced cell proliferation, migration, and anchorage-independent growth [53,54,55,56]. Similarly, we demonstrated increased G1-phase arrest and apoptosis which inhibited cell viability upon blockade of *CAMP*. *CAMP* promoted pancreatic ductal adenocarcinoma by activating cancer stem cell properties via a *P2X7*-dependent manner [57]. Additionally, *CAMP* knockdown downregulated stemness-related genes, as well as decreased production of oncospheres in SK-BR-3 breast cancer cells [58]. Similarly, we also observed a significant inhibition of ALDH activity, which is a well-known cancer stem cell marker, upon *CAMP* knockdown, which provides evidence that *CAMP* is stemness-related. 

It is well-established that macrophages secrete *CAMP* to kill target microbes [59]. During the differentiation from non-specific macrophage to M2 macrophage, we found that M2 marker expression was increased with *CAMP* expression and its receptor. To the best of our knowledge, this is the first study revealing the relationship between *CAMP* and TAMs in breast cancer. With limited studies on cathelicidin and M1/M2 macrophage polarization, LL-37 was reported to be inversely correlated with proinflammatory M1 macrophages [60]. Upregulation of *CD163, CAMP*, *P2X7* in macrophages cultured in conditioned medium or co-cultured with cancer cells strongly suggested a positive feedback loop between TAMs and breast cancer cells. The time-dependent increase in *CAMP* expression in the tumor microenvironment provides a novel targeted therapeutic option by blocking the positive feedback loop. There are different approaches targeting TAMs as to limit monocyte recruitment into tumor tissues by *CCL2*-blocking agent [61], to inhibit the activation of TAMs by *CSF1/CSF1R* inhibitors [62], reprogramm TAMs into antitumor macrophages using *CD40* agonist [63] or *IL-10* mAbs [64], and deplete macrophages by clodronate-liposome [65]. However, macrophage-targeting strategies using antibodies with large molecular weight often reduced the efficiency for tissue penetration while small molecule inhibitors tend to be less specific with increased risk of toxicity [66]. 

## 5. Conclusions

Taken together, *CAMP* was regarded as a target mediator in the cell–cell interaction between TAMs and cancer cells. Blockade of *CAMP* by either siRNA or antagonist reversed the upregulated M2 macrophage marker. In our in vivo experiment, the *CAMP* receptor antagonist indeed attenuated the TAM-associated tumor growth promotion effect, providing evidence that *CAMP* could serve as a potential target to inhibit breast cancer growth by disrupting the interaction between TAM andcancer cells. Based on our findings, *CAMP* targeted therapy aimed to intervene with the crosstalk between TAM andcancer cells mediated by *CAMP*, hoping to minimize the impact on the innate macrophage defensive function, especially for those with high circulating LL-37 titer.

## Figures and Tables

**Table 1:** Clinical characteristics of breast cancer patients.

	Breast Cancer (*n* = 110)
Age (years; mean (SD))	49.5 (12.8)
Histological type	
DCIS	38
IDC	72
Bilateral cancer	6
Stage	
0	41
I	34
II	25
III	6
IV	4
Histological grade	
1	8
2	34
3	21
NA	47

Abbreviation: DCIS—ductal carcinoma in situ; IDC—invasive ductal carcinoma.
